# Supplementary material for: MetaQTL: a package of new computational methods for the meta-analysis of QTL mapping experiments
Source: BMC Bioinformatics. 2007 Feb 8;8:49. doi: 10.1186/1471-2105-8-49 (PMC1808479; doi:10.1186/1471-2105-8-49)
Supplement: Additional File 7 — MetaQTL Package : jar file and tutorial. This Zip archive contains both the MetaQTL JAR file and the files of the tutorial. [file 1471-2105-8-49-S7.zip › org.inra.metaqtl/doc/org/metaqtl/algo/EMAlgorithm.html]

EMAlgorithm


|  |  |  |
| --- | --- | --- |
| **Overview** \| **Package** \| **Class** \| **Use** \| **Tree** \| **Deprecated** \| **Index** \| **Help** | |  |
| **PREV CLASS**   **NEXT CLASS** | **FRAMES**    **NO FRAMES**     **All Classes** |  |
| SUMMARY: NESTED \| FIELD \| CONSTR \| METHOD | DETAIL: FIELD \| CONSTR \| METHOD |  |


---


## org.metaqtl.algo Class EMAlgorithm

```
java.lang.Object
  org.metaqtl.algo.EMAlgorithm
```

---

``` public final class EMAlgorithm extends java.lang.Object ```

This class defines methods to perform the EM-algorithm
for univariate mixture of gaussians with known variances.

---

| **Field Summary** | |
| --- | --- |
| `static boolean` | `DO_SEM`             If true the best starting point is used to do Supplemental EM (SEM) in order to compute the DM Matrix.. |
| `static int` | `EM_CONTINUE`             The EM convergence status : continue |
| `static double` | `EM_ERR`             The EM convergence tolerance. |
| `static int` | `EM_FAILURE`             The EM convergence status : failure |
| `static int` | `EM_ITER_MAX`             The EM maximum number of iterations. |
| `static double` | `EM_MIN_DISTANCE`             The minimal mahalanobis distance between components. |
| `static int` | `EM_OK`             The EM convergence status : success |
| `static int` | `EM_START`             The number of replicates for a run of the EM-algorithm. |
| `static double` | `TINY_Z_PROBA`             The minimal value for Z proba in EM-clustering |


**Constructor Summary**

`EMAlgorithm()`


| **Method Summary** | |
| --- | --- |
| `static void` | `computeCOV(double[] sd, EMResult theta)`             Computes the variance-covariance matrix of the mixture component estimates. |
| `static void` | `computeDM(double[] x, double[] sd, double[] mu, EMResult theta)`             Computes the matrix of derivatives of the update function. |
| `static EMResult` | `doEM(double[] x, double[] sd, int k, EMResult spoint)`             Apply the EM-Algorithm on the given data set where `x` is an array of observed value and `sd` an array of same size than `x` which stores the standard deviations of the observed values. |
| `static void` | `emRate(EMResult theta, double[] mu, double[] pi)`             Compute the euclidean distance between the new parameters and the old ones to obtain a simple approximation of the EM convergence rate. |
| `static void` | `eStep(double[] x, double[] sd, EMResult theta)`             The Expectation step : it just consists in updating the Z matrix, i.e the cluster membership probabilities. |
| `static void` | `initEM(double[] x, double[] sd, EMResult theta, java.util.Random rng)`             Initializes the EM algorithm by randomly assigning observations to the clusters and do one M-Step to compute the first values of the parameters. |
| `static int` | `iterate(double[] x, double[] sd, EMResult theta)`             This method performs one iteration of the EM-algorithm. |
| `static int` | `mStep(double[] x, double[] sd, EMResult theta, double err)`             The Maximization step : here this step is straighforward and simple analytical formula are applied to obtain the new parameter estimates. |
| `static void` | `updateLoglikelihood(double[] x, double[] sd, EMResult theta)`             Updates the loglikelihood value. |
| `static double[]` | `updateMuVector(double[] x, double[] sd, EMResult theta)`             Updates the vector of mixture components and returns the new values. |
| `static double[]` | `updatePiVector(EMResult theta)`             Updates the vector of mixture mixings and returns the new values. |
| `static void` | `updateZMatrix(double[] x, double[] sd, EMResult theta)`             Updates the Z matrix. |

**Methods inherited from class java.lang.Object**

`clone, equals, finalize, getClass, hashCode, notify, notifyAll, toString, wait, wait, wait`

**Field Detail**

### EM\_START

```
public static int EM_START
```

:   The number of replicates for a run of the EM-algorithm.

---


### EM\_ITER\_MAX

```
public static int EM_ITER_MAX
```

:   The EM maximum number of iterations.

---


### EM\_ERR

```
public static double EM_ERR
```

:   The EM convergence tolerance.

---


### EM\_MIN\_DISTANCE

```
public static double EM_MIN_DISTANCE
```

:   The minimal mahalanobis distance between
    components.

---


### DO\_SEM

```
public static boolean DO_SEM
```

:   If true the best starting point is used to
    do Supplemental EM (SEM) in order to
    compute the DM Matrix..

---


### TINY\_Z\_PROBA

```
public static final double TINY_Z_PROBA
```

:   The minimal value for Z proba in EM-clustering

    **See Also:**: Constant Field Values

---


### EM\_OK

```
public static final int EM_OK
```

:   The EM convergence status : success

    **See Also:**: Constant Field Values

---


### EM\_CONTINUE

```
public static final int EM_CONTINUE
```

:   The EM convergence status : continue

    **See Also:**: Constant Field Values

---


### EM\_FAILURE

```
public static final int EM_FAILURE
```

:   The EM convergence status : failure

    **See Also:**: Constant Field Values


**Constructor Detail**

### EMAlgorithm

```
public EMAlgorithm()
```


**Method Detail**

### doEM

```
public static EMResult doEM(double[] x,
                            double[] sd,
                            int k,
                            EMResult spoint)
```

:   Apply the EM-Algorithm on the given data set where `x`
    is an array of observed value and `sd` an array of same
    size than `x` which stores the standard deviations of
    the observed values. The underlying mixture model will have
    `k` distinct components and its parameter estimates
    will be returned as an `EMResult`. If `soint`
    is not null then the EM will start at the given point specified by
    the mixture parameters of `soint`. To control some
    behaviours of the EM-algorithm use the global variables of the class.

    :   **Parameters:**: `x` - the observed positions.: `sd` - the standard deviation of the observed positions.: `k` - the number of mixture components.: `spoint` - the starting point for the EM-algortihm **Returns:**: a `EMResult` from which the parameter estimates can be obtained. **See Also:**: `EMResult`

---


### initEM

```
public static void initEM(double[] x,
                          double[] sd,
                          EMResult theta,
                          java.util.Random rng)
```

:   Initializes the EM algorithm by randomly assigning observations to
    the clusters and do one M-Step to compute the first values of the
    parameters.

    :   **Parameters:**: `x` - the observed values.: `sd` - the standard deviations of the observed values.: `theta` - the current parameter estimates.

---


### iterate

```
public static int iterate(double[] x,
                          double[] sd,
                          EMResult theta)
```

:   This method performs one iteration of the EM-algorithm. An
    iteration consists in two steps :

    - Expectation (E-step) : where the expectation of the
      cluster membership missing variable is computed.
    - Maximization (M-step) : where the parameters are updated
      to the values which maximize the expectation.

    If the iteration leads to an update of the parameters which is under
    the suited range of convergence error then the method will return
    `EM_OK, otherwise it will return EM_CONTINUE.
    In some cases it can happen - mainly due to numerical roundoff errors -
    that the likelihood value after the iteration is lower than the likelihood
    before the iteration which in theory cannot happen. Then in this case the
    method will return EM_FAILURE.

    :   Parameters:: x - the observed values.: sd - the standard deviations of the observed values.: theta - the current parameter estimates. Returns:: the status of the iteration.`

---


### eStep

```
public static void eStep(double[] x,
                         double[] sd,
                         EMResult theta)
```

:   The Expectation step : it just consists in updating the
    Z matrix, i.e the cluster membership probabilities.

    :   **Parameters:**: `x` - the observed values.: `sd` - the standard deviations of the observed values.: `theta` - the current parameter estimates. **See Also:**: `updateZMatrix(double[], double[], EMResult)`

---


### mStep

```
public static int mStep(double[] x,
                        double[] sd,
                        EMResult theta,
                        double err)
```

:   The Maximization step : here this step is straighforward and
    simple analytical formula are applied to obtain the new parameter
    estimates. The method returns the status of the maximization.
    If the maximization leads to an new value of the parameters which is under
    the suited range of convergence error then the method will return
    `EM_OK, otherwise it will return EM_CONTINUE.
    In some cases it can happen - mainly due to numerical roundoff errors -
    that the likelihood value after the maximization is lower than the likelihood
    before the iteration which in theory cannot happen. Then in this case the
    method will return EM_FAILURE

    :   Parameters:: x - the observed values.: sd - the standard deviations of the observed values.: theta - the current parameter estimates. See Also:: updateMuVector(double[], double[], EMResult), EMAlgorithm#updatePiVector(double[], double[], EMResult), updateLoglikelihood(double[], double[], EMResult)`

---


### emRate

```
public static void emRate(EMResult theta,
                          double[] mu,
                          double[] pi)
```

:   Compute the euclidean distance between the new parameters and the
    old ones to obtain a simple approximation of the EM convergence
    rate.

    :   **Parameters:**: `theta` - the current parameter estimates.: `mu` - the last estimates of the means.: `pi` - the last estimates of the mixing.

---


### updateMuVector

```
public static double[] updateMuVector(double[] x,
                                      double[] sd,
                                      EMResult theta)
```

:   Updates the vector of mixture components and returns the new
    values.

    :   **Parameters:**: `x` - the observed values.: `sd` - the standard deviations of the observed values.: `theta` - the current parameter estimates. **Returns:**: the new vector of means as an array of double.

---


### updatePiVector

```
public static double[] updatePiVector(EMResult theta)
```

:   Updates the vector of mixture mixings and returns the new
    values.

    :   **Parameters:**: `theta` - the current parameter estimates. **Returns:**: the new vector of mixings as an array of double.

---


### updateZMatrix

```
public static void updateZMatrix(double[] x,
                                 double[] sd,
                                 EMResult theta)
```

:   Updates the Z matrix.

    :   **Parameters:**: `x` - the observed values.: `sd` - the standard deviations of the observed values.: `theta` - the current parameter estimates.

---


### updateLoglikelihood

```
public static void updateLoglikelihood(double[] x,
                                       double[] sd,
                                       EMResult theta)
```

:   Updates the loglikelihood value.

    :   **Parameters:**: `x` - the observed values.: `sd` - the standard deviations of the observed values.: `theta` - the current parameter estimates.

---


### computeDM

```
public static void computeDM(double[] x,
                             double[] sd,
                             double[] mu,
                             EMResult theta)
```

:   Computes the matrix of derivatives of the update
    function. This is done by applying one iteration
    of the EM algorithm to a sligh modified vector of
    the current parameter estimates.

    :   **Parameters:**: `x` - the data points.: `sd` - the standard deviation of the data points.: `mu` - the previous estimate of the means: `theta` - the current parameter estimates.

---


### computeCOV

```
public static void computeCOV(double[] sd,
                              EMResult theta)
```

:   Computes the variance-covariance matrix of the
    mixture component estimates. If the global
    variable `DO_SEM` has been
    set to false then the observed information won't be
    computed and the variance-covariance matrix will be
    obtained by using only the complete information.

    :   **Parameters:**: `sd` - the standard deviations.: `theta` - the maximum likelihood estimate.


---


|  |  |  |
| --- | --- | --- |
| **Overview** \| **Package** \| **Class** \| **Use** \| **Tree** \| **Deprecated** \| **Index** \| **Help** | |  |
| **PREV CLASS**   **NEXT CLASS** | **FRAMES**    **NO FRAMES**     **All Classes** |  |
| SUMMARY: NESTED \| FIELD \| CONSTR \| METHOD | DETAIL: FIELD \| CONSTR \| METHOD |  |


---
